# Control Centre for Intensive Care as a Tool for Effective Coordination, Real-Time Monitoring, and Strategic Planning During the COVID-19 Pandemic

**DOI:** 10.2196/33149

**Published:** 2022-02-16

**Authors:** Martin Komenda, Vladimír Černý, Petr Šnajdárek, Matěj Karolyi, Miloš Hejný, Petr Panoška, Jiří Jarkovský, Jakub Gregor, Vojtěch Bulhart, Lenka Šnajdrová, Ondřej Májek, Tomáš Vymazal, Jan Blatný, Ladislav Dušek

**Affiliations:** 1 Institute of Biostatistics and Analyses Faculty of Medicine Masaryk University Brno Czech Republic; 2 Institute of Health Information and Statistics of the Czech Republic Prague Czech Republic; 3 Department of Simulation Medicine Faculty of Medicine Masaryk University Brno Czech Republic; 4 Ministry of Health of the Czech Republic Prague Czech Republic; 5 Clinic of Anaesthesiology, Perioperative and Intensive Medicine Masaryk Hospital in Ústí nad Labem Ústí nad Labem Czech Republic; 6 General Staff Czech Armed Forces Prague Czech Republic; 7 Clinic of Anaesthesiology, Resuscitation and Intensive Medicine University Hospital in Motol Second Faculty of Medicine of Charles University Prague Czech Republic; 8 Department of Paediatric Haematology and Biochemistry University Hospital Brno Brno Czech Republic

**Keywords:** COVID-19, coronavirus, intensive care, inpatient care, online control center, prescription, open data, ICU, monitoring, strategy, development, app, function, Czech Republic, inpatient, crisis management

## Abstract

In the Czech Republic, the strategic data-based and organizational support for individual regions and for providers of acute care at the nationwide level is coordinated by the Ministry of Health. At the beginning of the COVID-19 pandemic, the country needed to very quickly implement a system for the monitoring, reporting, and overall management of hospital capacities. The aim of this viewpoint is to describe the purpose and basic functions of a web-based application named “Control Centre for Intensive Care,” which was developed and made available to meet the needs of systematic online technical support for the management of intensive inpatient care across the Czech Republic during the first wave of the pandemic in spring 2020. Two tools of key importance are described in the context of national methodology: one module for regular online updates and overall monitoring of currently free capacities of intensive care in real time, and a second module for online entering and overall record-keeping of requirements on medications for COVID-19 patients. A total of 134 intensive care providers and 927 users from hospitals across all 14 regions of the Czech Republic were registered in the central Control Centre for Intensive Care database as of March 31, 2021. This web-based application enabled continuous monitoring and decision-making during the mass surge of critical care from autumn 2020 to spring 2021. The Control Center for Intensive Care has become an indispensable part of a set of online tools that are employed on a regular basis for crisis management at the time of the COVID-19 pandemic.

## Introduction

The COVID-19 pandemic has placed an urgent burden on health care systems, communication among their components, and health care management. Although the majority of patients have a mild or even asymptomatic course of the disease, approximately 8% to 15% of patients require hospitalization during the infection or afterward [1]. Approximately 21% to 36% of patients hospitalized due to COVID-19 are admitted to the intensive care unit (ICU) [2-4], and the average mortality of those admitted to the ICU is approximately 30% [5,6].

In the majority of developed countries, ICU beds are often close to their full capacity even under normal circumstances, let alone during the COVID-19 pandemic. Modeling studies suggest that a pandemic of severe influenza or another similar disease would require ICU and mechanic ventilation capacity that is significantly greater than what is available and that many patients who would require a ventilator might not have access to one [7,8].

By definition, epidemics and disasters will result in many patients arriving in a continuous stream, with shortages of necessary technology, beds, oxygen support, ventilators, medications, as well as trained health care personnel. The high likelihood of such conditions emphasizes the importance of having a framework that is suitably constructed to allow users to anticipate and adapt to these inevitable complexities and challenges [8]. Beds, equipment, and medications should be monitored ideally in “real time” and electronically, with knowledge of both their absolute numbers and whereabouts immediately available to system leaders [9]. For example, ventilatory support is an absolute necessity for the survival of critically ill patients and may be the single most important therapy that dictates the outcomes. Moreover, ventilators are highly demanding for staff qualification; they are therefore likely to be the limiting factor in any hospital’s ability to accommodate a large surge of mass critical care [10]. The COVID-19 pandemic has generated a sense of urgency that will allow the adoption of innovation without the logistical barriers and path dependencies that we have become accustomed to. Web-based solutions can fill a critical gap for the allocation of health care resources [11].

The Czech National Control Centre for Acute Inpatient Care (CNCC-AIC) was established under the auspices of the Ministry of Health of the Czech Republic in May 2020 to provide strategic data-based and organizational support for individual regions and for providers of acute care at the nationwide level. Its main purpose is to monitor hospital capacities, to provide analytical reporting on these capacities, and to enable the overall management of health care facilities run by all inpatient care providers in the Czech Republic.

The operative online tools of key importance employed by the CNCC-AIC involve the Control Centre for Intensive Care (CC-IC) [12], an online database updated on a daily basis, which provides an overview of capacities of inpatient care (classified into categories of acute, long-term, and supportive care) and a dedicated “Clinic” module, which is part of the Information System of Infectious Diseases (a database of people with laboratory-confirmed COVID-19).

In accordance with the defined methodology, the CC-IC was brought into operation in April 2020 to enable the continuous monitoring as well as daily reporting of available data on the occupancy rate of inpatient beds. The aim of this viewpoint paper is to present the methodology of managing capacities in Czech hospitals during the ongoing COVID-19 pandemic. A newly developed and robust technical solution, namely the CC-IC, is also introduced. The methodological background of the design and implementation of this unique practical operative tool, which is built on the long-term experience of experts across the Czech health care system, and experience with its practical use are presented.

## Structure of the CC-IC

### Overview

The CC-IC is an entirely new platform that has been developed since the start of the COVID-19 pandemic. This has been achieved in mutual cooperation among the following groups of involved stakeholders and experts to assess recent progress of the epidemic and recommend measures for crisis situations caused by the COVID-19 epidemic in the Czech Republic: (1) a team of regional coordinators of intensive care; (2) the Integrated Central Management Team, which includes representatives from the Ministry of Health, Czech Armed Forces, National Institute of Public Health, National Agency for Communication and Information Technologies, and Institute of Health Information and Statistics of the Czech Republic; and (3) a team of developers from a joint workplace of the Institute of Health Information and Statistics of the Czech Republic and the Institute of Biostatistics and Analyses at the Faculty of Medicine of Masaryk University.

The CC-IC provides two tools of key importance to offer effective support for the CNCC-AIC: (1) a module for regular online updates and overall monitoring of currently free capacities in hospitals (health care technology/medical devices, beds, staff) in real time, and (2) a module for online entering and overall record-keeping of requirements on medications for COVID-19 patients. No patient personal data or medical records are stored in the CC-IC application; thus, no anonymization or pseudonymization algorithms are used.

The CC-IC also specifically monitors the reprofiled capacity (ie, beds that had previously, under normal circumstances, in a given health care facility) been intended for the provision of care of another type or another specialty.

**Figure 1:**
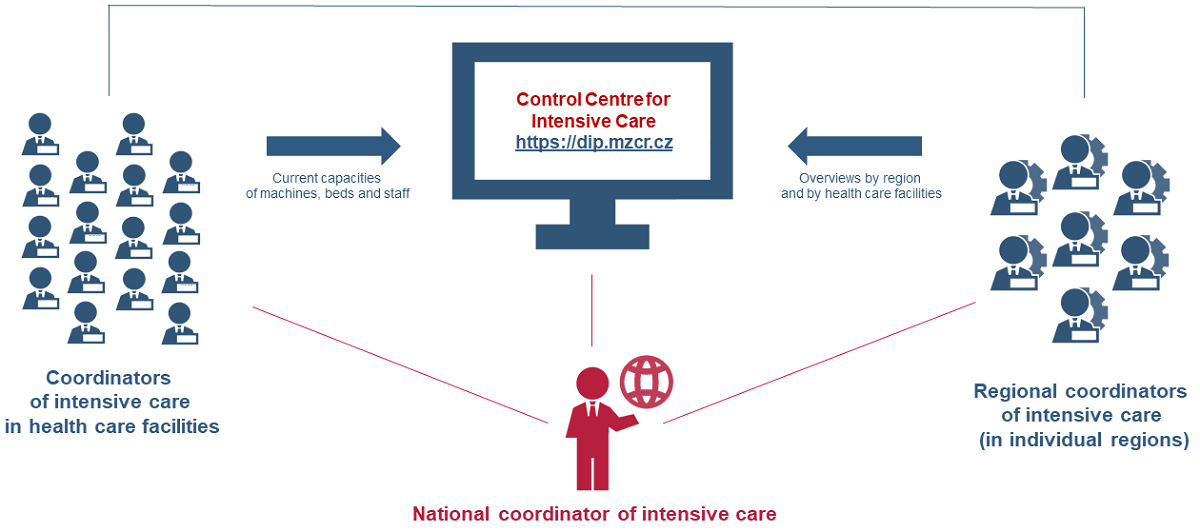
Schematic diagram of the Control Centre for Intensive Care (CC-IC).

The CC-IC is a web application that has been continuously developed and run by the Institute of Health Information and Statistics of the Czech Republic, which cooperates closely with the Ministry of Health of the Czech Republic. Since the very beginning, the development has been based on requirements and needs for the coordination of intensive care, as required by health care facilities in the Czech Republic (Figure 1).

Use of the application (available in the Czech language only) relies on several basic principles: (1) user authentication and authorization, (2) definition of user roles and rights to edit and read, (3) each new user registration is approved by the CNCC-AIC, and (4) private and free email addresses are not allowed. CC-IC users include representatives of individual health care facilities (management, coordinators of intensive care, physicians, and nurses), emergency medical service workers, pharmacy workers, and members of the Integrated Central Management Team.

**Figure 2:**
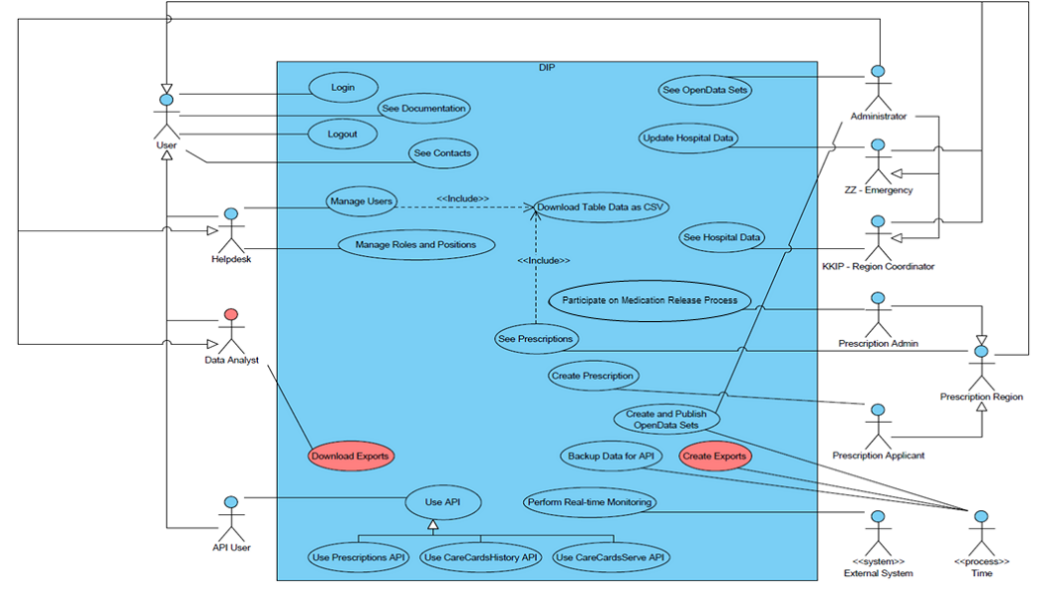
Use case diagram for the Control Centre for Intensive Care system. API: application programming interface.

The use case diagram in Figure 2 provides an overview of the entire CC-IC system, including key functions assigned to users in specific roles. The main objective of this diagram is to depict the various actors as well as interactions that are available to these actors.

Use cases provide insight into the basic structure of CC-IC functional requirements. Based on modeling of individual use cases, which are associated with the primary actors, we can see individual parts of the system so that we can decompose and divide the whole system into separate submodules. Moreover, the CC-IC provides an authenticated application programming interface (API) for secure sharing of selected data sets, which consists of access to a list of requirements on medications and an export of currently free capacities in hospitals, including their history.

### Regular Monitoring of Health Care Providers and the ICU Network

On April 9, 2020, the Ministry of Health of the Czech Republic adopted an exceptional measure, ordering all acute care providers to use the CC-IC to immediately report any changes in capacities of intensive care related to COVID-19 patients. Since the launch of the CC-IC, the availability of free capacity of machines, beds, and staff has been monitored on a regular basis (Textbox 1). Hospitals have been requested to report their intensive care capacities on a daily basis, usually at the end of the day. The system allows for continuous updates at any time; more frequent entering of the latest data would nevertheless bring an unnecessary burden to hospital staff at the time of a significant shortage of capacities in terms of personnel, time, and equipment.

Based on data in the CC-IC, it is possible to coordinate the provision of inpatient care centrally, on a nationwide level. In each individual region, hospitalizations of patients who need admission to an intensive care facility (ICU and/or emergency department) are managed and coordinated by the so-called regional coordinator of intensive care, who is a physician specialized in anesthesiology and intensive care medicine. These physicians cooperate closely with regional emergency medical services and with representatives of regional authorities. Apart from that, each health care facility has prepared emergency beds (ie, inpatient beds that could be used to provide care for COVID-19 patients if all existing capacities are occupied).

The system reacts to immediate needs by individual regions, depending on the current occupancy rate of machines and beds, combined with the number of COVID-19 patients. If an increase in the number of new patients is detected within the CC-IC, it is necessary to prepare health care facilities for a crisis situation. This involves limiting capacities for patient admissions (ie, only admit patients who require intensive and emergency care, and suspend the provision of nonemergency care); discharging as many patients as possible to home care; plan an increased bed supply in health care facilities for an increase in COVID-19 patients; and provide a sufficient number of competent health care professionals.

The module for regular online updates and overall monitoring of the currently free capacities of intensive care in real time is predominantly used by coordinators of intensive care in individual health care facilities. A total of 134 intensive care providers from all 14 regions of the Czech Republic are registered in the central CC-IC database. As of March 31, 2021, a total of 927 users were registered who used this module to update data on intensive care capacities in predefined intervals (ie, at least once a day during critical periods of the COVID-19 pandemic), who made 86,565 sessions in total.

**Figure 3:**
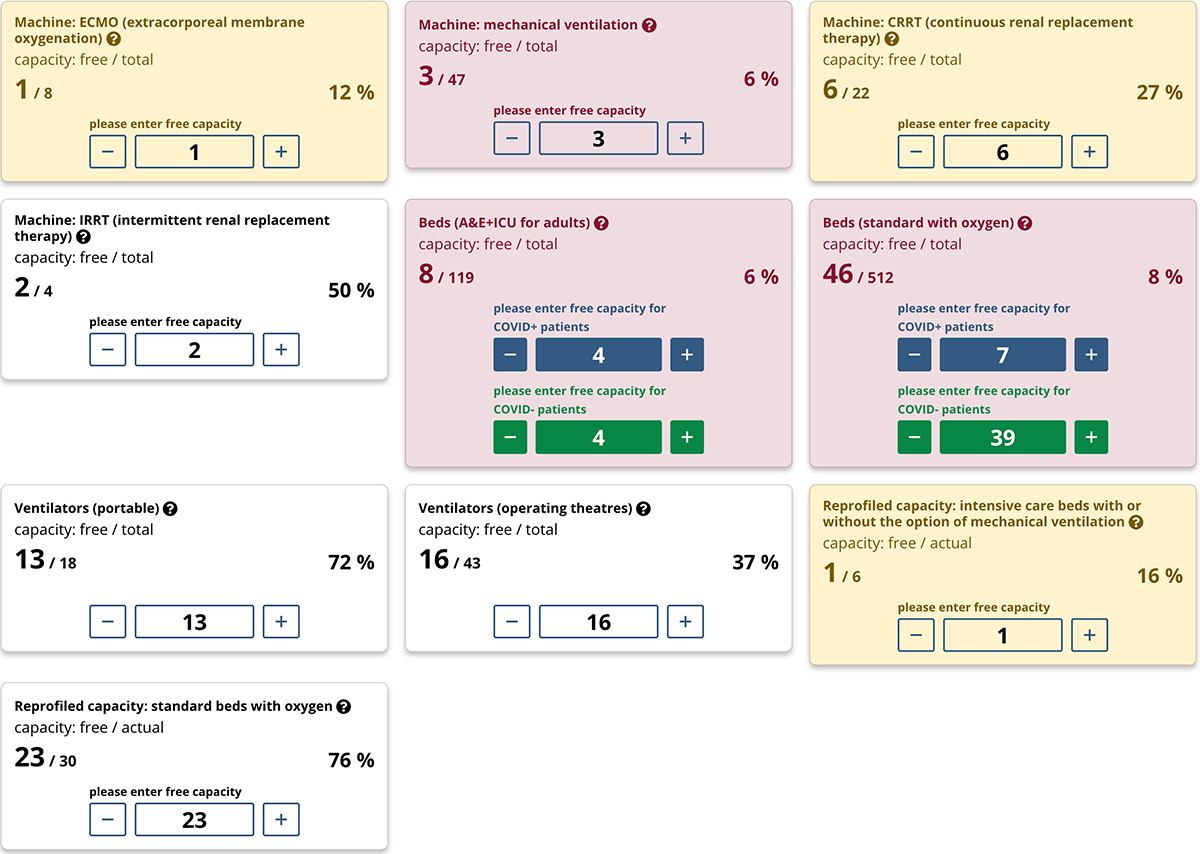
Interface for updates of free capacities. Overview of intensive care capacities for the capital, Prague, as of December 30, 2020. A&E: accident and emergency; ICU: intensive care unit.

A user-friendly and simple interface (Figure 3) is available to users, who can therefore update occupancy rates of available capacities (in terms of machines, beds, and staff) very easily. The entire application is fully responsive and therefore entirely compatible with all types of devices, namely smartphones, tablets, laptops, and desktops. Free capacities for COVID-19–positive versus COVID-19–negative patients are distinguished only for beds. Entering a free bed into the system means that this bed is fully functional (ie, all required equipment and corresponding health care professionals are available). Monitoring of staff and equipment in and out of service has turned out to be ineffective and unsustainable on a long-term basis; this function was therefore removed from the system upon the decision of the Central Integrated Management Team. Across the entire module, each individual card has one of three background colors to provide a quick overview of free capacities: white, <30% to 100%> free capacity; yellow, <10% to 30% free capacity; and red, <0% to 10% of free capacity.

**Overview of monitored information on capacities of health care facilities providing inpatient care in the Czech Republic.**

**Health care technology/medical devices**

- Extracorporeal membrane oxygenation
- Mechanical ventilation in accident and emergency (A&E) departments and in intensive care units (ICUs) for adults
- Continuous renal replacement therapy
- Intermittent renal replacement therapy
- Transport ventilators
- Anesthesia machine with mechanical ventilator

**Hospital beds**

- A&E and ICU beds for adults; a distinction is made between beds for COVID-19–positive and COVID-19–negative patients
- Standard beds with oxygen; a distinction is made between beds for COVID-19–positive and COVID-19–negative patients
- Reprofiled capacity: beds with or without the option of ventilatory support devices

**Staff**

- Physicians (A&E and ICU for adults)
- Nurses (A&E and ICU for adults)

Two different reports are available to provide a quick overview of the latest data. These reports are primarily used by regional coordinators of intensive care, members of the operational team of the CNCC-AIC, members of management of individual health care facilities, and top officials of the Ministry of Health of the Czech Republic. The first report is a summary data table providing a clearly arranged view of all health care facilities, including real-time absolute and relative occupancy numbers as regard to available capacities. The second is an aggregated visualization in the form of context cards, which also show real-time absolute and relative occupancy numbers in regard to available capacities, but users can further choose among the nationwide view, views for individual regions, or those for individual health care facilities.

The example screenshot from the CC-IC application in Figure 3 also demonstrates an aggregated visualization of key parameters monitored in real time for the capital, Prague. Four cards have a yellow background because the available capacity of corresponding parameters was reported to be below 30% at the time.

**Figure 4:**
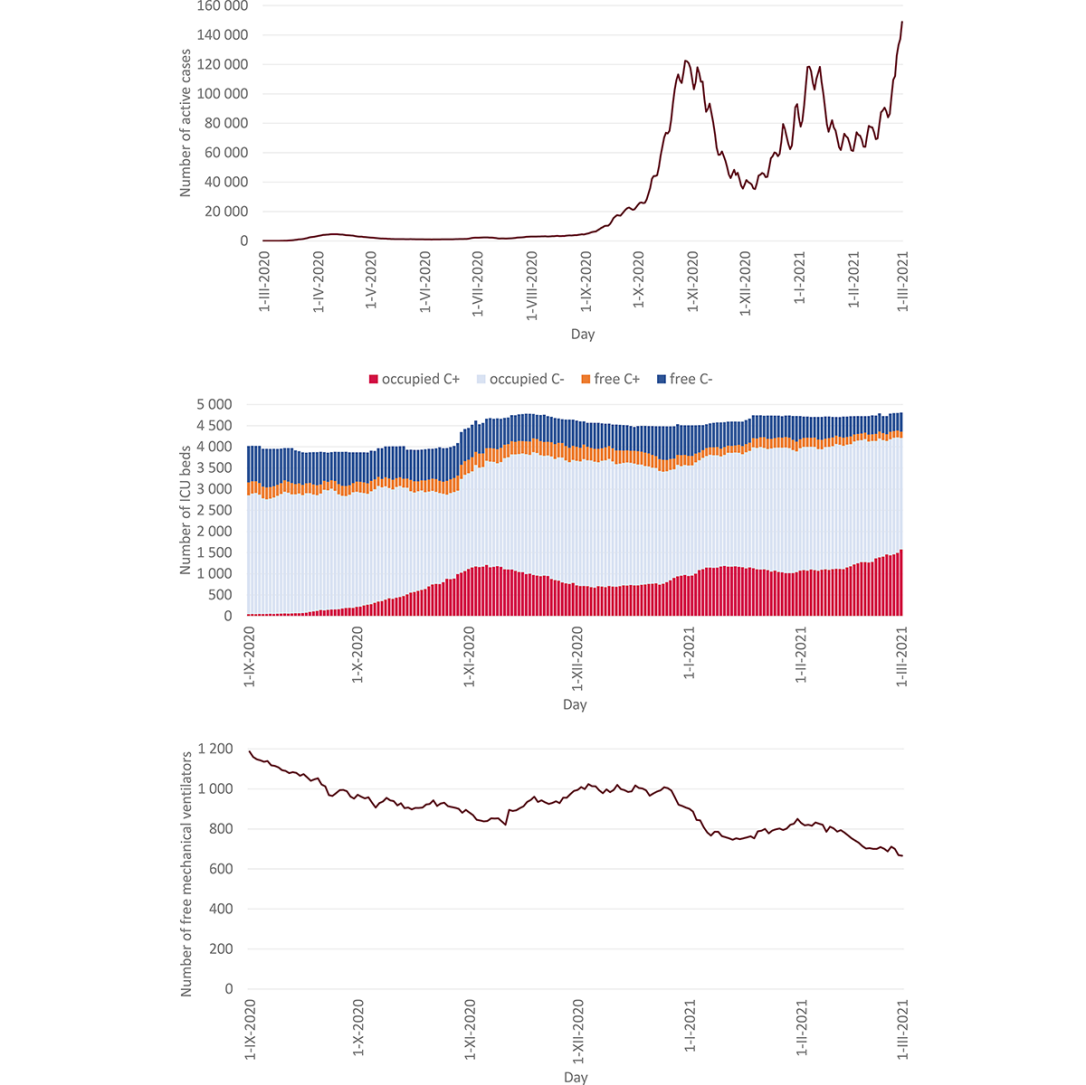
Time trends of COVID-19 cases and related intensive care, from top to bottom: number of active cases (prevalence), occupied and free intensive care unit (ICU) beds for COVID-19 patients and other patients, and availability of mechanical ventilators.

Owing to the timely and strict measures at the national level, the first COVID-19 wave in the spring of 2020 did not bring significant pressure to hospital-based care, with a maximum daily prevalence of around 4500 active cases and less than 100 ICU beds being occupied with COVID-19 patients among the Czech population of over 10 million people. The situation changed in the autumn of 2020; the number of active cases peaked twice to approximately 120,000, first in early November 2020 and then in early January 2021 (Figure 4, top). The number of patients requiring intensive care at the ICU fluctuated at around 1200 in these periods. Requirements for intensive care increased to an even more critical level in the second half of February 2021, with 1574 ICU beds occupied by COVID-19 patients on March 1, 2021 (Figure 4, middle).

The increasing numbers of hospitalized patients with COVID-19 during October 2020, as steadily monitored by the CC-IC, required increased capacities of standard beds and ICU beds. The total numbers of both types of beds were increased by repurposing the existing beds and adding new beds, according to the capabilities of individual hospitals. The availability of mechanical ventilators as an essential equipment of intensive care followed the overall trends: it decreased from nearly 1200 machines on September 1, 2020, to less than 700 at the end of February 2021 (Figure 4, bottom). If a temporary depletion of ICU capacities occurred in some regions, patients were transferred to hospitals in other regions in which free capacities were available according to the CC-IC report. Over 100 patients were transferred between hospitals during autumn 2020 and winter 2021, usually from small local hospitals to large hospitals in regional capitals or in Prague. All of these decisions were taken by the national and regional intensive care coordinators in cooperation with the Central Integrated Management Team.

### Prescription of COVID-19 Medications

With an increasing number of hospital admissions of COVID-19 patients who need medication to manage the disease, it was necessary to secure the systematic record-keeping and distribution of these medications to individual health care facilities. For this reason, a dedicated module was developed within the CC-IC, making it possible to request any of three therapies against COVID-19: remdesivir, convalescent plasma, and favipiravir. The tool has been intentionally designed in a rather flexible manner so that it can be easily adapted and employed after the end of the pandemic, thus providing a generally applicable ordering system for any medication.

The CC-IC enables physicians from any involved health care facility to request a certain medication for a specific patient. The module has three basic user roles: (1) applicant for medication (physician), which has been assigned to an approved group of registered users from individual health care facilities who are thus entitled to enter new requests from their respective health care facility; (2) regional coordinator of intensive care, who is entitled to enter new requests from health care facilities from across the entire region and can also see an overview of requests not only from the region but from the Czech Republic as a whole; (3) virtual indication group, with members entitled to approve requests for medications from the respective region and they can also see an overview of requests from across the Czech Republic; and (4) contact persons from pharmacies linked to specific health care facilities, who are entitled to supply the approved medications.

Mutual cooperation among representatives of the Ministry of Health of the Czech Republic, regional coordinators of intensive care, members of the virtual indication group, and representatives of pharmacies led to the development of a structured form that is employed to send a request for a specific medication. The form consists of three parts: (1) health care facility, containing information on the physician and hospital requesting the medication; (2) specification of therapy, containing information on the requested medication and the date of supply; and (3) patient description, which is an anonymized description of the patient’s condition and their risk factors (without mentioning any personal data in conformity with personal data protection according to General Data Protection Regulation).

**Figure 5:**
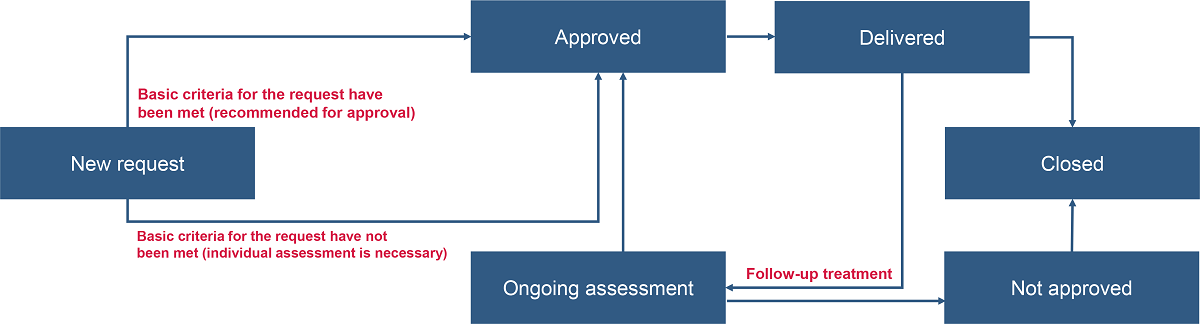
Description of the life cycle of a request for a medication in the Control Centre for Intensive Care (CC-IC).

After the form is filled out and sent, validation of individual items is performed within the CC-IC, the request is stored in the central database, and a notification is sent to all users involved (physician, member of the virtual indication group in the respective region, pharmacy). The medication must be explicitly approved before being supplied by a respective pharmacy that secures supplies for a given health care facility. In the final step, the request is closed for the purpose of archiving and subsequent statement of charges for the respective health insurance company. In exceptional cases, request for follow-up treatment can occur after the administration of remdesivir; such requests can also be processed by the CC-IC. The life cycle of a request for a medication within the CC-IC is shown in Figure 5.

All health care facilities providing acute care began to report, by means of the CC-IC, the current use of these capacities on April 9, 2020, when the first version of the system was launched. The first version only contained one module, which enabled regular online updates and overall monitoring of currently free capacities of intensive care in real time. On June 10, 2020, the second module was launched for the online entering and overall record-keeping of requirements on medications for COVID-19 patients.

A major added value of the entire CC-IC system is the fact that it was designed to meet the current needs of acute medicine in response to the unexpected burden on inpatient care capacity caused by the COVID-19 pandemic. The data that the CC-IC allows to collect provide a key support for data-oriented decision-making processes within ICU occupancy crisis management. Thanks to the CNCC-AIC and a proper methodological setup, it is possible to effectively coordinate, for example, elective care, which can have a negative effect on patients’ health when being moved or postponed. The following paragraphs briefly describe the functions of both of the above-mentioned modules as well as analytical reports and open data sets that have been provided as an outcome of the CC-IC database.

As of December 30, 2020, a total of 4759 requests for medication were registered in the CC-IC, out of which 4666 (98.05%) were requests for remdesivir. The remaining 93 (1.95%) requests were for convalescent plasma. On that date, a total of 496 users entitled to work with requests for medications were registered in the system; these users included physicians in individual health care facilities, regional coordinators of intensive care, pharmacy workers, and members of the virtual indication group. Between the first wave and the second wave of the COVID-19 pandemic, there was a major change in the functioning of the module for the sending and record-keeping of requests for medications. A new version was implemented in September 2020, fully supporting the entire process of requests for medications (entering, validation of entered data, sending, approval/rejection, supply, conclusion), including email notifications. The form itself for sending a request contains a list of key information necessary for subsequent approval and the supply of a specific medicine (see Multimedia Appendix 1).

## Practical Use of Open Data

Data on free capacities are available as records in the National Catalogue of Open Data [13,14] in a regularly updated data set that describes two waves of the COVID-19 pandemic in the Czech Republic.

The data set contains overviews of machine equipment (extracorporeal membrane oxygenation, mechanical ventilation, continuous renal replacement therapy, intermittent hemodialysis, ventilators—portable and those in operating theaters) and occupancy rate of inpatient beds (only beds in the emergency department and ICU for adults and standard beds with oxygen in the entire hospital). Data are regularly updated for beds suitable for the provision of the requested type of care (ie, necessary staff as well as corresponding material and technical equipment must also be available). The definition of the necessary number of staff for a given type of bed can be different among individual hospitals. These data have been available since September 1, 2020, when the obligation for hospitals to update free capacity was reset to a 1-day interval.

On top of that, for the purpose of the transfer of record-based (nonaggregated) data directly from the CC-IC to the Institute of Health Information and Statistics of the Czech Republic analytical team and to external authorized systems (run by the leadership of the Capital of Prague, for example), an API was developed to provide a machine-readable alternative to user access. For a secure transfer of data, each user must be authenticated and authorized, and the following conditions must be met: (1) each new user of the API must first register in the CC-IC system and must be explicitly approved for a given user role; (2) each request must contain the so-called API key (api_key), which is an integral part of the query (query string); (3) other parameters can also be part of the query string parameter, which are listed at specific endpoints; and (4) for each user, there is a defined list of allowed endpoints, IP addresses, and parameter values. If the user’s request does not pass through the authorization process, the user will obtain the response “401 Unauthorized.”

Data from the CC-IC, along with information from other registries (eg, the Information System of Infectious Diseases), are regularly processed for the purpose of daily reporting, which is provided to the team of the CNCC-AIC. Among dozens of analytical outputs (as of December 23, 2020), one of them monitors—on a daily basis—the situation in the Czech Republic from the points of view of currently hospitalized patients, new admissions to hospitals, new discharges from hospitals, as well as the utilization of health care facilities in individual regions, expressed by their free capacity [15].

## Discussion

The Czech Republic was one of the most affected countries during the COVID-19 pandemic waves in autumn 2020 and winter 2021 in terms of incidence, hospitalizations, and deaths. The CC-IC was one of the key tools developed to manage the capacities of standard and ICU beds in the entire country, and to ensure that appropriate care was provided not only to patients with COVID-19 but also to other patients.

The Czech Republic has by far the highest density of ICU beds (43.2 beds per 100,000 population) and one of the longest average hospital stays (9.5 days) among the Organization for Economic Cooperation and Development countries [16]. There is also a high level of centralization of hospital-based care, because all large hospitals are under the control of either the Ministry of Health or regional authorities. These factors enabled a very quick response and management of intensive care, including the compulsory reporting of current capacities into the central system.

The spread of COVID-19 has usually not been uniform, and there have been different ICU demands within the affected countries and their regions. Central reporting systems for the monitoring of ICU capacities were therefore established in some countries [17]. The absence of such a platform, often combined with a fragmented health care system and small ICU facilities, may slow down the response and hold back the provision of appropriate care to critically ill patients, both with and without COVID-19 [18].

The systems for ICU capacities monitoring employed in various countries are usually operated under the auspices of national authorities (ie, government, ministries, and armed forces). They have been designed to provide near real-time data for crisis management and decision-making [19-21]. Short- and long-term estimates of ICU bed occupancy in the upcoming days and weeks using statistical models are another important output [21,22].

The CC-IC platform has been designed and developed in a tailor-made manner, responding to needs of the leadership of the Ministry of Health of the Czech Republic and crisis teams that are actively involved in the management of the COVID-19 epidemic in the Czech Republic. Based on the need to quickly and effectively resolve various crisis situations related to the capacity of ICUs, the CC-IC system was incorporated into the Czech legislation. Experience with the COVID-19 pandemic has clearly shown the requirement for long-term monitoring of ICU bed occupancy. This is the main reason why the obligation to update the occupancy on a daily basis for all health care facilities providing inpatient care in the Czech Republic is now enshrined in the Czech legislation. As part of international cooperation, the possibility of transfer of this online tool to other countries is being discussed. With respect to the general concept of the CC-IC design, the transferability of this tool to other countries should be trouble-free provided that the basic methodology for capacity monitoring in health care facilities is adhered to. It is of key importance that new requirements are collected from health care professionals themselves, even in the future. Intensivists should be part of strategic planning committees before, during, and after pandemics to coordinate ICU responses with hospital and regional efforts for triage, clinical care, and infection control [7]. Regular evaluation includes the analysis of user behavior and further optimization of the whole platform, so that it can continue to function as the primary support tool for all involved stakeholders.

### Conclusions

The web platform CC-IC has been developed as a reaction to the urgent need of strategic data-based and organizational support for individual regions and providers of acute care on a nationwide level. The platform is intended for online real-time reporting of changes of free capacity of hospitals, real-time reporting of occupancy rate and availability of beds, a clearly arranged reporting of health care facilities, and the basis for quick decision-making of crisis management during the COVID-19 pandemic. Delegated representatives of health care facilities (coordinators of intensive care) are tasked with reporting the remaining free capacity of machines and beds that are available for patients hospitalized with COVID-19. Information on newly reported current capacity is immediately available to the Integrated Central Management Team, which continuously monitors the situation across the Czech Republic. Online entering and nationwide record-keeping of requests for medications intended for COVID-19 patients are processed by a standalone module. This system can be accessed by users from individual health care facilities providing acute care; the users typically involve several physicians and management representatives from the same health care facility, so that they can stand in for each other and share the latest information. Development of the CC-IC is far from complete; the system has been further improved, partly based on requirements by members of the team of the CNCC-AIC and partly in response to suggestions by users themselves.

## Appendix

Multimedia Appendix 1 Key information necessary for subsequent approval of a specific medication.

## Abbreviations

API: application programming interface
CC-IC: Control Centre for Intensive Care
CNCC-AIC: Czech National Control Centre for Acute Inpatient Care
ICU: intensive care unit
