# Supplementary material for: Control Centre for Intensive Care as a Tool for Effective Coordination, Real-Time Monitoring, and Strategic Planning During the COVID-19 Pandemic
Source: J Med Internet Res. 2022 Feb 16;24(2):e33149. doi: 10.2196/33149 (PMC8852654; doi:10.2196/33149)
Supplement: Multimedia Appendix 1 [file jmir_v24i2e33149_app1.docx]

# Multimedia Appendix 1: Key information necessary for subsequent approval of a specific medication

- Name and surname of the person requesting the medication
- Name and surname of the responsible supervisor
- Name and type of the workplace indicating the medication
- Requested medication
- Is the indication for administration of the requested medication demonstrably mentioned in medical records? (yes/no)
- Date of supply
- Patient’s category of therapy (low-flow oxygen therapy, high-flow oxygen therapy, non-invasive ventilation, ECMO)
- Patient’s age
- Date of onset of clinical symptoms
- Date of result of the PCR test
- Patient’s risk factors (lymphocytopenia, haemato-oncological malignancy, vasculitis or vasculopathy, obesity with BMI > 30, diabetes mellitus type 2, hypertension, chronic obstructive pulmonary disease, another risk factor)
- Is the indication in accordance with the Summary of Product Characteristics (SPC)? (yes/no)
- Is there bilateral pulmonary damage described by a radiologist or a pulmonary specialist? (yes/no)
- In view of the patient’s current condition, can restoration of integrity of organ functions be expected? (yes/no)
- Has the patient’s long-time prognosis been evaluated as favourable? (yes/no)
- Based on the “frailty score”, the patient’s functional status corresponds to ... (select on the scale from 1 to 6)
- Comment on the patient (optional)
